# Is the Age of Novel Ecosystem the Factor Driving Arbuscular Mycorrhizal Colonization in *Poa compressa* and *Calamagrostis epigejos*?

**DOI:** 10.3390/plants10050949

**Published:** 2021-05-10

**Authors:** Gabriela Woźniak, Damian Chmura, Eugeniusz Małkowski, Paulina Zieleźnik-Rusinowska, Krzysztof Sitko, Barbara Ziemer, Agnieszka Błońska

**Affiliations:** 1 Institute of Biology, Biotechnology and Environmental Protection, Faculty of Natural Sciences, University of Silesia in Katowice, 28 Jagiellońska Str., 40-032 Katowice, Poland; gabriela.wozniak@us.edu.pl (G.W.); eugeniusz.malkowski@us.edu.pl (E.M.); paulina.zeleznik-rusinowska@us.edu.pl (P.Z.-R.); krzysztof.sitko@us.edu.pl (K.S.); 2 Institute of Environmental Protection and Engineering, University of Bielsko-Biała, 2 Willowa Str., 43-309 Bielsko-Biała, Poland; dchmura@ath.bielsko.pl; 3 Silesian Botanical Garden in Miokołów, 5 Sosnowa Str., 43-190 Mikołów, Poland; basia.ziemer@gmail.com

**Keywords:** mycorrhiza colonization, vegetation development, spontaneous succession, non-analogous species assemblages, novel ecosystems

## Abstract

Some sites transformed or created by humans (novel ecosystem) are different both in vegetation and ecosystems establishment and development. The unknown habitat conditions and new species composition is resulting in new abiotic and biotic systems. To improve the understanding of the process governing the relationships between the environmental factors, plant species assemblages and their arbuscular mycorrhizal fungi (AMF) inoculation were studied in chronosequence on post-coal mine heaps. We hypothesized that AMF root colonization will be dependent on the age of heap and not on the dominant plant species (vegetation type). The high frequency of mycorrhizal colonization of roots (F%) of *Poa compressa-* and *Calamagrostis epigejos*-dominated vegetation type was stated. All mycorrhizal parameters were lower in *C. epigejos* roots when compared to *P. compressa* (ranging from 60% to 90%). The highest relative mycorrhizal intensity, M%, and mean abundance of arbuscula, A%, in the roots of both examined plants were recorded in vegetation patches dominated by *Daucus carota*. Positive and statistically significant correlations were found between F%, M%, and A%, and lack of correlation between the heaps’ age and mycorrhizal parameters, and statistically significant correlations between A% and potassium and magnesium content were revealed. The interspecific relations in the novel ecosystems become more complex along with the increase of diversity.

## 1. Introduction

Four features have described the formal definition of the concept of “novel ecosystem”. The origins of a novel ecosystem are dependent on human agency, and the ecological thresholds have to be crossed for its emergence, leading to significantly altered (non-analogues) species composition. A novel ecosystem possesses the capacity to sustain itself, and they exist without historical precedents, so it is impossible to reclaim the novel ecosystems to the “previous” stage [1]. The above definition brings a completely new perspective to the environmental role of sites transformed by human activity. It facilitates the use of new practical solutions in management applications, based on the still-developing knowledge about the environmental functioning under intense human pressure. Traditional approaches emphasizing historical continuity are challenged by the novel ecosystem concept that can deliver critical ecosystem services and are immune to most global change impacts [2,3,4,5]. The post-coal mine heaps fulfill the four requirements of the novel ecosystems definition and consist of a model example of the developing novel ecosystem [6].

Some of the not reclaimed post-industrial sites, on which the spontaneous vegetation and ecosystem development are going on, can provide a unique opportunity to study the relations between plants and other organisms such as AMF in the novel ecosystems field conditions. According to Wang [7], who reviewed the large number of studies focused on AMF’s role during the recovery of mining-impacted sites, the majority of studies have been conducted as greenhouse experiments. Only very few results have been obtained from the fieldwork performed in spontaneous vegetation patches. The presented study is an example of research focused on the relation between vascular plant species and parameters of AMF colonization in respect to the spontaneous variety of biotic (vegetation type) and abiotic substrate conditions performed in the field in non-analogous vegetation communities of the novel ecosystem.

A characteristic feature of the non-analogous vegetation growing on these post-coal mine heaps is the mosaic of vegetation patches dominated by various species confined to a variety of microhabitats. It is created by species typical of aquatic and marsh habitats, as well as dry meadow, stony gravel communities, and ruderal habitats. For the vegetation growing in post-industrial areas, the most precise diagnosis of the vegetation type is the dominant plant species, which very distinctively determines the physiognomy and functionality of the vegetation patch [8,9]. Dominant plant species, in terms of their percentage cover abundance, can be accompanied by a large group of species with varied cover abundance [10]. Consequently, the dominant plant vegetation characteristic was applied in this paper to choose the most frequent vegetation types occurring on heaps of different age.

Among the biotic properties, plant–microbe interactions in the rhizosphere are primary determinants of plants, plants’ assemblages, and soil substrate health and development [11,12,13,14,15,16,17]. Major beneficial soil microbiota components are mycorrhizal fungi [14,18,19,20] suggested that colonization of roots by AMF could be important for plants colonizing the post-coal mine heaps. It has been stated that mycorrhizae contribute to plant growth and help in successful colonization of, e.g., mineral habitats [20,21] and survival [18,19,22,23,24,25]. Firstly, mycorrhizal fungi improve plant nutrition [19,21,24,25] through transformation of insoluble nutrients forms into bioavailable forms [19,21]. Secondly, they alleviate the water deficiency of plants due to increased water uptake from the soil with the help of hyphae [24,25]. Thus, mycorrhizae could determine the rate of plant succession [18,26], especially when the soil has low concentrations of available nutrients, mostly phosphorus [25], and low water-holding capacity [19,26].

The relationship between the biotic and abiotic elements is particularly complex and important in new developing ecosystems on sites changed or created by humans, such as post-industrial sites [27,28,29,30,31]. The studies which are covering the interactions between different groups of organisms (plants, bacteria, and fungi) are less frequent [5,16,17,32,33]. Recently, it has been emphasized that understanding of the mechanisms which govern the spontaneous vegetation development in environmental conditions created by humans is of crucial importance for efficient urban-industrialized areas’ management [1,24,34,35]. It is particularly important for sites for which the knowledge about the ecosystem and environmental functioning from the natural or semi-natural habitats cannot be used [10,36,37,38]. On sites heavily transformed or created by humans—novel ecosystem—the ecosystems develop in a different non-analogous way [39] Hobbs et al. 2006; [40].

Most of the studies focused on AMF’s plant relationships have been conducted on reclaimed, topsoil brown coal open-cast and lead-zinc smelter sites, oriented on a few separated plant species in an experimental greenhouse design. Only very few results have been obtained from the fieldwork study [7]. Our study material was collected based on a unique long-term study conducted on un-reclaimed spontaneous vegetation patches development. The analyzed plant individuals have been obtained from vegetation patches, which are the indicators of particular chronosequence ecosystem development. In this respect, we believe that our study provides a new quality to the survey of novel ecosystem functioning.

The aim of the present study was to investigate the level of root colonization by arbuscular mycorrhizal fungi in *Poa compressa* and *Calamagrostis epigejos* in spontaneous vegetation patches on post-hard coal mine heaps in relation to (i) age of heap, (ii) the substrate properties, and (iii) the dominant plant species using a chronosequence approach. We hypothesized that arbuscular mycorrhizal fungi root colonization will not be dependent on the dominant plant species (vegetation type), but rather on the age of heap and/or the heap substrate properties. In the nutrient-poor, severe conditions of the post-coal mining heaps, the environmental filters play the most important role in shaping the relations between organisms, while in nutrient-rich habitats, the relations between organisms are more dependent on intra- and inter-species competition. In detail, we expect that positive relations between heap age, trophy, and fungi root colonization, i.e., values of mycorrhization indices, should increase with increasing content of potassium, magnesium, nitrogen, and age.

## 2. Materials and Methods

### 2.1. Study Site Description

The hard coal mine heaps provide harsh, mineral substrate environmental conditions for plant colonization and growth [19,41,42], with such physico-chemical properties as: lack of soil, low fertility, low water-holding capacity [10,19], variable pH, high salinity, high temperatures [10], vulnerability to erosion, and high compaction of substrate material [43]. The post-hard coal mine heaps are sites of a pure mineral substrate and can temporarily be extremely warm (50 °C at noon in summer) compared with their surroundings, but with no differences in precipitation. The study was carried out on post-hard coal mine heaps located in the Silesian Upland (southern Poland). The climate is temperate with a mean annual precipitation of ca. 580 mm and a mean annual temperature of 7.6 °C. Coal mine heaps are usually built of carboniferous gangue with unfavorable substrate texture (mainly clay stone and siltstone, also sandstone, conglomerate, coal shale) with small admixtures of coal [10]. The research plots have been established on a few heaps: “KWK Wesoła” (50°10′30″ N, 19°05′40″ E), “KWK Murcki” (50°11′20″ N, 19°02′02″ E), “KWK Wieczorek” (50°12′51″ N, 19°04′19″ E), and “KWK Sośnica” (50°16′17″ N, 18°44′36″ E). 

The vegetation patches with the occurrence of the studied plant species where the roots for the AM sample have been obtained had to fulfill all of the following prerequisites: (i) representing the most common vegetation type for the particular heap age class, and (ii) in the chosen vegetation patch, at least ten individuals of the studied plant species have to be present. In order to fulfill the first prerequisite, the stratified random sampling approach has been adopted, as described in Piekarska-Stachowiak et al. [44]. The studied heaps were divided into four age classes (Class I—up to 10 years old; II—10–30 years old; III—30–60 years old; IV—over 60 years old), based on the information obtained in the previous study performed on factors influencing the spontaneous vegetation diversity (1998–2007) by Woźniak [10]. As a result of this long-term study, it was found that one of the most frequent dominant species in age Class I was *Poa compressa*, while *Calamagrostis epigejos* and *Daucus carota* were very common among the vegetation on heaps of age Class II. In the vegetation patches of the older heaps (Class III and IV), the dominant plant species were trees such as *Betula pendula* and *Pinus sylvestris*. 

The presented study focused on two plant species: *Poa compressa* and *Calamagrostis epigejos*, because apart from being the dominants on the spoil heaps of age Class I and II, these two plant species frequently occur as companion species in vegetation patches on spoil heaps of the Class III and IV (Table 1). The result of the long-term preliminary study provided the basis for random selection of the vegetation patches where the roots of the studied plants were obtained [10]. Each of them had to fulfil the second prerequisite. In each of the selected vegetation patches, ten individuals of the studied plant species have been sampled for assessing the arbuscular mycorrhizal fungi colonization. The total number of analyzed individuals depends on the number of selected patches in the particular heap’s age class.

The *Poa compressa* and *Calamagrostis epigejos* individuals for which the level of root colonization by arbuscular mycorrhizal fungi has been assessed and the rhizosphere substrate samples that were collected in selected vegetation patches growing spontaneously on post-hard coal mine heaps were not technically reclaimed. Plant and soil samples were collected in relation to vegetation types and the different age classes of heaps, and the scheme of the experiment is presented in Table 1. The individuals of *Poa compressa* and *Calamagrostis epigejos* species were present in all studied vegetation patches, which reflected the habitat conditions and the co-occurring species’ influence along the chronosequence of the novel ecosystem’s development on post-hard coal mine heaps. The list of species and their abundances (percentage cover assessed by eye) of plant species present in each of the 4 m × 4 m vegetation patches were recorded (data not shown). Soil substrate and root samples of ten plant individuals were taken from each studied vegetation patch.

### 2.2. Soil Sampling and Analyses

Soil substrate samples consisting of 5 soil sub-samples (depth 0–15 cm of the studied plant’s root system) were collected from each of the selected vegetation patches. The five soil substrate sub-samples from each patch were mixed, air-dried at 30 °C, and sieved to the 2 mm fraction prior to soil physico-chemical analyses. Soil moisture content was determined by using the gravimetric method: difference between 10 g wet and dry weights (105 °C for 24 h) of soil samples. Soil pH in 1 M KCl and electrical conductivity (EC) in distilled water were measured after 24 h of equilibration (ratio 1:2.5 m/v) [45]. Available potassium was analyzed following Dobrzański et al. [45] and available magnesium using the Schatschabel method [45]. The total phosphorus and organic carbon were measured using the colorimetric and the Tiurin method, respectively. The ammonium nitrogen (N-NH4) and nitrate nitrogen (N-NO3) content were measured separately following the methods in Fotyma et al. [46].

### 2.3. Assessment of Mycorrhizal Colonization

The evaluation of mycorrhizal status was performed on individuals of two plant species: *Poa compressa* and *Calamagrostis epigejos*. From each of the 33 vegetation patches that fulfilled the requirements of the field chronosequence experiment, the root samples of ten plant individuals from each patch have been collected during the plants’ flowering time. For mycorrhizal investigations, the thinnest, almost the last, parts of lateral roots were used. After washing in tap water, the roots were prepared using the modified method of Phillips and Hayman [47]. The roots were softened in 10% KOH for 24 h at room temperature, rinsed in water, acidified in 5% lactic acid for 24 h at room temperature, and stained with 0.01% aniline blue in 5% lactic acid for 24 h at room temperature. The stained roots were stored in lactoglycerol until they were used for slide preparation. From the roots of each of the collected plants, thirty 1 cm long root sections were analyzed. Mycorrhizal parameters were assessed according to Trouvelot et al. [48]: mycorrhizal frequency (F%), describing the frequency of mycorrhizal roots in the root system, relative mycorrhizal root length (M%), showing the intensity of colonization in the whole root system, and relative arbuscular abundance (A%), describing the arbuscule richness in the root system.

### 2.4. Statistical Analyses

Prior to statistical analyses, all the data were verified for the assumptions of normality using Shapiro–Wilk’s test and variance homogeneity by means of the Levene test. When it was possible, parametrical tests were applied. In the next step, one-way analyses of variance (ANOVAs) were performed to examine significance of differences in root colonization parameters (F%, M%, A%) among distinguished patches characterized by dominant plant species for *Calamagrostis epigeios* and *Poa compressa,* separately. Since we had a relatively small dataset, we applied permutational ANOVA (package LmPerm). When ANOVA yielded significant results, then the LSD Fisher test for multiple comparisons was performed. The Venn diagrams were constructed to disentangle the effect of the most significant variables. They show partition of explained variance among constrained variables. Adjusted coefficient of determination for RDA was by means of the *RsquareAdj()* function implemented in the vegan package. A probability of 0.05, or less, was considered to be statistically significant. In order to assess the influence of soil variables and parameters of heaps, vegetation as well as effect of species, i.e., *Calamagrostis epigeios* and *Poa compressa* on root colonization, redundancy analysis (RDA) was performed using the vegan package. Prior the analysis, detrended correspondence analysis (DCA) was run in order to show the main directions of vegetation succession along age of waste heaps. The first two axes of DCA were considered as proxies of vegetation succession due to chronosequence of vegetation patches. In RDA, the stepwise selection of environmental variables based on *p*-value and AIC criterion was performed. The procedure of forward selection was carried out. Both soil variables and parameters of waste heaps: age and the first two axes of DCA, were included in the analysis. Data were processed by using R language and environment [49].

## 3. Results

### 3.1. Substrate Properties

The substrate was acidic, and its pH varied from 4.60 to 6.75. It contained up to 6.60 mg 100 g^−1^ of P, 19.50 mg 100 g^−1^ of K, 32.50 mg 100 g^−1^ of Mg, 13.80 mg 100 g^−1^ of NH4+, and 18.7% of organic carbon. The high level of organic carbon has been recorded due to the presence of geogenic carbon, which originates from particles of hard coal from the tertiary strata. Despite the high total organic carbon content, the tested soils were poor in accessible carbon sources. The content of nitrate nitrogen was very low, and in all samples, it was below 1 mg 100 g^−1^ (Table 2).

### 3.2. Poa compressa

**Figure 1:**
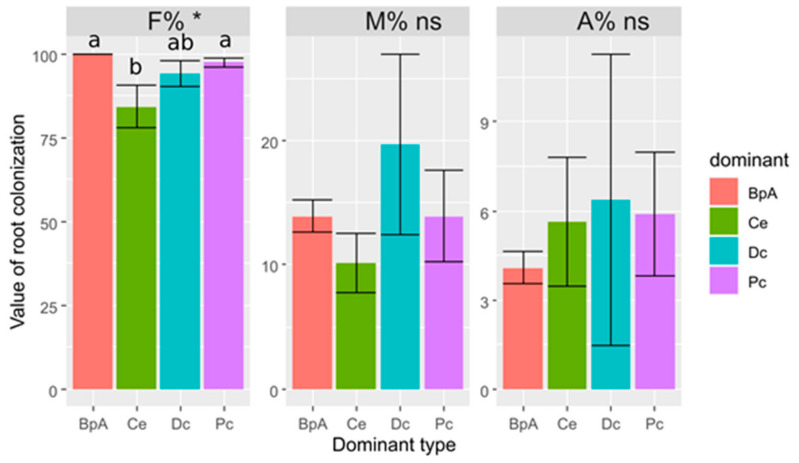
The mycorrhizal parameters (Mean ± SE) in the root system of *Poa compressa* in the vegetation patches dominated by Pc—*Poa compressa*, Ce—*Calamagrostis epigejos*, Dc—*Daucus carota*, and BpA—*Betula pendula* tree individuals. Results of ANOVA: ns—non-significant, * *p* < 0.05. The various letters above bars denote the significance of differences at *p* < 0.05 after LSD test.

The frequency of arbuscular mycorrhizal colonization (F%) of *Poa compressa* roots was very high in patches where *Betula pendula* and *P. compressa* were dominant (*Betula pendula A*). In contrast, the lowest mycorrhizal frequency was recorded in the roots of *P. compressa* in patches dominated by *Calamagrostis epigejos* (Figure 1). The intensity of the mycorrhizal colonization (M%) in the root system of *P. compressa* was medium and ranged from 10% to 20%. The lowest value of M% was recorded in the roots of *P. compressa* in the patches dominated by *C. epigejos*, and the highest was in the patches dominated by *Daucus carota*, however the differences were not statistically significant (Figure 1). The arbuscular abundance (A%) in the root system of *P. compressa* was low and did not differ significantly, irrespective of dominant plant species (Figure 1).

### 3.3. Calamagrostis epigejos

**Figure 2:**
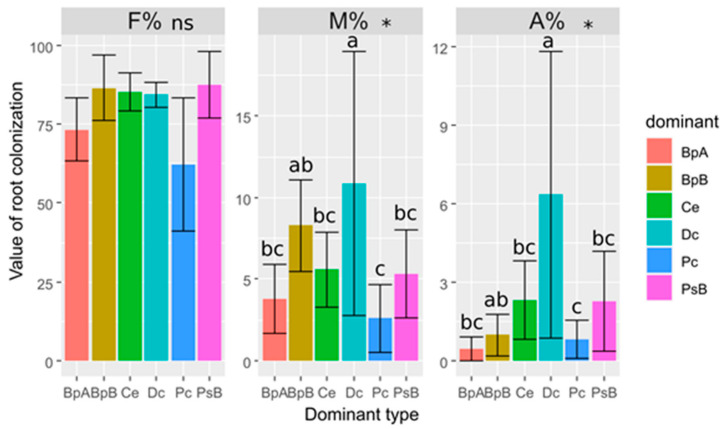
The mycorrhizal parameters (Mean ± SE) in the root system of *Calamagrostis epigejos* in the vegetation patches dominated by Pc—*Poa compressa*, Ce—*Calamagrostis epigejos*, Dc—*Daucus carota*, BpB—*Betula pendula* shrub, BpA—*Betula pendula* tree, and PsB—Pinus sylvestris shrub. Results of ANOVA: ns—non-significant, * *p* < 0.05. The various letters above bars denote significance of differences at *p* < 0.05 after LSD test.

All mycorrhizal parameters were lower in *C. epigejos* roots when compared to *P. compressa*. The frequency of mycorrhizal colonization of roots of *C. epigejos* ranged from about 60% for patches dominated by *P. compressa* to 90% for patches dominated by *Pinus sylvestis* shrubs (*Pinus sylvestis* B (PsB)) patches, but the differences were not statistically significant. The highest value of the intensity of mycorrhizal colonization (M%) was recorded in the roots of *C. epigejos* growing in vegetation patches dominated by *Daucus carota*, and it was significantly higher in comparison with M% of *C. epigejos* collected from the vegetation patches dominated by *P. compressa* and tree *Betula pendula A* (BpA). Similar to M%, the arbuscular abundance (A%) was the highest in the roots of *C. epigejos* in the patches dominated by *Daucus carota* and it was significantly higher when compared to A% in the patches dominated by *P. compressa*, *C. epigejos,* and *Betula pendula* trees, as well as *Betula pendula* shrub (BpB). It is also important to note that the arbuscular abundance (A%) in the roots of *C. epigejos* in the patches dominated by *Daucus carota* was higher than in patches where *C. epigejos* was the dominant plant (Figure 2).

### 3.4. Ordination Results

The obtained results demonstrated that contents of magnesium and potassium as well as age of heap and first axis of DCA significantly contributed to variance of root colonization. The species identities of mycorrhizal species, i.e., *C. epigejos* and *P. compressa*, were not significant (Table 3).

**Figure 3:**
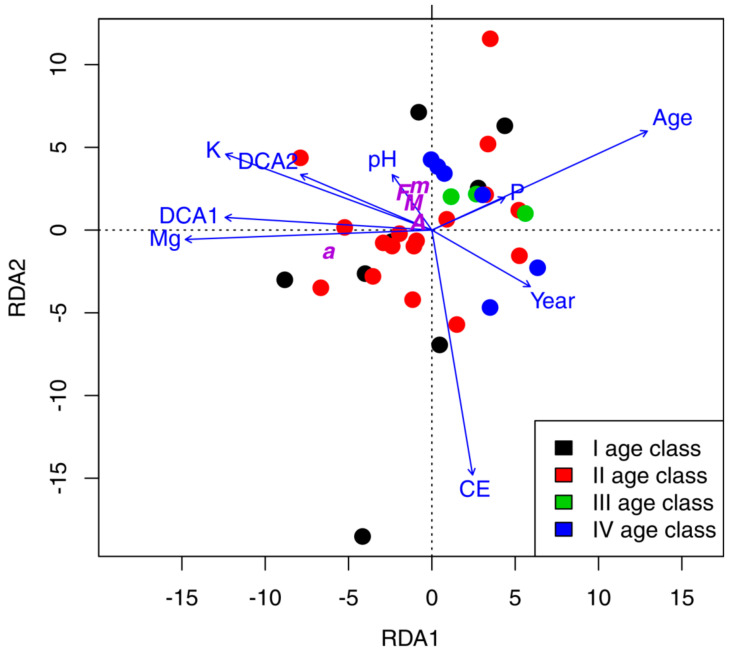
Tri-plot of RDA based on mycorrhizal parameters showing samples in four classes of age (1–4) and environmental factors.

The contents of both nutrients were correlated negatively with the first axis of RDA; likewise, vegetation gradient was expressed as the first two axes of DCA, while age of waste heap was correlated positively (Figure 3).

**Figure 4:**
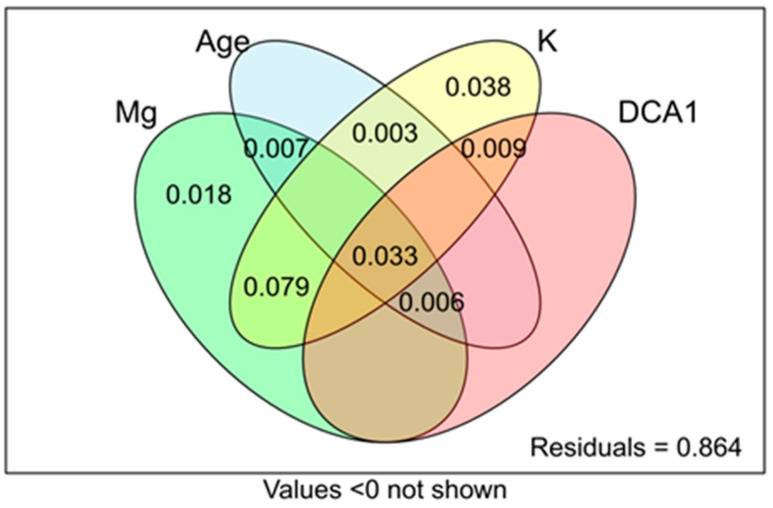
The Venn diagrams showing variance partitioning among RDA constraints.

The F%, M%, m%, A%, and a% were correlated, except for a%, which was distinct. The Venn diagrams showed that environmental variables explained only 13.6% of variation of mycorrhizal parameters (Figure 4).

The highest adjR2 was found for K (0.15), followed by Mg (0.13). The age of heap and DCA1 (vegetation gradient) were less important, scoring only 0.019 and 0.012 of adjR2 values, respectively.

## 4. Discussion

The present study was focused on the assessment of the level of root colonization by arbuscular mycorrhizal fungi in individuals of two species, *Poa compressa* and *Calamagrostis epigejos.* The analyzed individuals were collected from different (i) substrate properties and (ii) co-occurring species assemblages. Both factors were changing with time, which was comprehensively reflected in the vegetation patches, which were the indicators of a particular chronosequence ecosystem development stage. According to [50] Rydlová et al. (2016), little is known about the relations between the plants growing in particular vegetation communities and the AM fungal communities during primary succession on the human reshaped substrate. In their study, Rydlová et al. [50] performed an experiment using four age groups’ substrates from brown-coal strip mine heaps.

The root colonization by arbuscular mycorrhizal fungi in the studied spontaneous vegetation patches were lower in *C. epigejos* roots when compared to *P. compressa*: up to 90% for patches representing later successional stages, dominated by tree *Pinus sylvestis* (PsA) shrubs (*Pinus sylvestis* B (PsB)). There was no correlation between the age of the heaps and mycorrhizal parameters of the studied plants. The F% mycorrhizal parameters of *P. compressa* and *C.* *epigejos* individuals growing in vegetation patches of the first and last successional stage were the highest. The A% and M % for *P. compressa* and *C.* *epigejos* individuals were the highest in vegetation patches dominated by *Daucus carota* representing the second successional stage.

### 4.1. The Habitat Conditions and Age of the Site

The high frequency of mycorrhizal colonization of roots (F%) of *Poa compressa* and *Calamagrostis epigejos* indicated that these plants are able to establish AMF associations in patches dominated by different plant species. However, the low mycorrhizal frequency (M%) and arbuscular abundance in roots (A%) could mean that nutrient exchange between these plants and the resident AMF community is not substantial, or there is a lack of appropriate AMF species in the substrate. Such explanation for the observed relationships were proposed by Endresz et al. [51] for *Calamagrostis epigejos* and *Cynodon dactylon* species.

It has already been recorded that plants which grow on post-industrial habitats such brown coal topsoil become mycorrhizal in the early stages of plant succession [52], and arbuscular mycorrhiza is suggested to be particularly important for plants in the early successional stage in such sites [53,54], supporting the P phosphor deficiency. Püschel et al. [55] indicate that AMF affects the coexistence of plants being the dominant species, the community structure, and the progress in plant community structure within the succession development [56]. In general, in our study, all mycorrhizal parameters were lower for *Calamagrostis epigejos* compared to *Poa compressa*. In the study conducted on semiarid temperate vegetation in Hungary, *Calamagrostis epigejos* individuals showed low dependence on arbuscular mycorrhiza, which was explained as an important contributory factor in its success in habitats with soil disturbance [57]. While in the study on post-brown coal open-cast mine heaps in Northern Bohemia, *Calamagrostis epigejos* was moderately colonized by mycorrhizal fungi and seemed to be facultatively mycorrhizal. Similarly, *Poa compressa* is well-adapted to nutrient uptake and has low mycorrhizal responsiveness. However, in terms of N deficiency, it was considerably colonized by AMF [58]. In general, some plants, because of numerous root hairs and a high nutrient uptake capability, are less dependent on mycorrhizae unless they grow on degraded soils and in soils with an extreme nutrient deficiency [52,59]. However, grasses of alien origin could be more dependent on fungi root colonization, e.g., *Avena fatua* [60]. In the case of native species, likewise in the present study, the process of adaptation to an occurrence in man-made habitats had a chance to run for a longer time, thus they are not so fungi-dependent.

Considering the influence of nutrient availability, it is generally accepted that AMF takes part in a nutrient exchange between fungi and plants in natural and managed habitat systems ([61,62,63]. This symbiosis is particularly important for the uptake by plants of P, N, and such micronutrients as Cu, Mn, or Zn [64,65]. However, in the present study, the statistically significant correlations were found only between arbuscular abundance and the content of K and Mg.

The results of Kasowska [66] on heaps from fireloam coal mining showed that mycorrhizal colonization of *Calamagrostis epigejos* had higher values of mycorrhizal parameters in the 8–9-year-old-stage of succession than in the 1–2-year-old. Gould et al. [67] obtained similar results but in a shorter period of time. They observed that on several managed mines’ surface banks, after the first year of plant succession, the mycorrhizal colonization was low, and in the second year, it increased significantly along with the vegetation diversity. They also observed that the density of propagules increased significantly. Thereafter, mycorrhizal parameters appeared to stabilize [67]. (Rydlová and Vosátka [52] suggested that the mycorrhizal colonization might rely on the mycotrophic status of plant species or on the abundance of mycorrhizal propagules in the soil or soil substrate. The summary of the main results of the study conducted on *Calamagrostis epigejos* and *Poa compressa* and AMF colonization is presented in Table 4.

### 4.2. The Influence of Co-Occurring and Dominant Plant Species

The species composition and spatial structure of the vegetation of post-industrial areas (e.g., post-hard coal mine heaps) is different from any known in natural or semi-natural sites, which makes it difficult to classify using the vegetation type based on accepted phytosociological methods [42,74]. Although, it would be expected that the studied vegetation will consist mainly of ruderal species coming from the urban-industrial environment. However, the long-term observations have revealed that the vegetation spontaneously developing on post-industrial sites consists of many different ubiquitous species with a wide ecological tolerance [9,75]. Mycorrhizal status of *Poa compressa* and *Calamagrostis epigejos* individuals differed depending on the dominant plant species in the patch. The highest M% and A% in the roots of both examined plants were in the patches dominated by *Daucus carota*. The effect of *Daucus carota* on mycorrhizal status of the examined species may be due to some flavonoid compounds in root exudates of *Daucus carota* which are able to stimulate the rate of spore germination and the hyphal growth of AM fungi [76]. For this reason, *Daucus carota* is used in production of AM fungus inoculum [77].

Significantly lower values of M% and A% were observed when compared to patches dominated by *Daucus carota* for *Calamagrostis epigejos* individuals with the individuals obtained from patches dominated by trees of *Betula pendula A* for parameter M%, and *Betula pendula A* and *B* for parameter A%. It is known that some trees promote ectomycorrhizal fungi at the expense of endomycorrhizal fungi, which reduce AMF colonization of neighboring plants relying on AMF. In a study presenting this phenomenon, *Salix caprea* growing on un-reclaimed heaps created by open-pit coal mining in the Czech Republic had a negative impact on understory cover and biomass (with *Poa compressa* and *Calamagrostis epigejos,* among others) through below-ground competition. A similar phenomenon of competitive effects of overstorey trees on under-storey herbs at restored sites was observed for *Populus tremula*, *Pinus halepensis,* and *Lonicera maackii* [78].

The mycorrhizal parameters F% and M% of *Poa compressa* were the lowest in vegetation patches dominated by *Calamagrostis epigejos*. This result is supported by the study of Endresz et al. [51], who indicated that the presence of expansive species, such as *Calamagrostis epigejos*, influences the degree of AMF colonization for sand vegetation dominant plant *Festuca vaginata,* and the parameters were lower compared to a natural community without *Calamagrostis epigejos*. It has been stated that *Calamagrostis epigejos* may alter the soil mycorrhizal community, which reduces the mycorrhizal colonization and subsequently the growth, reproduction, and abundance of resident species.

In our study, it has been stated that all examined species were mycorrhizal in every age class (including the first age class). A lack of correlation between the age of the heaps and mycorrhizal parameters of the studied plant species has been stated. The results obtained in the present study might suggest that the interspecific relations developing in the novel ecosystems of post-hard coal mine heaps are more complex with the diversity increase and do not follow the simple age-dependent relation.

### 4.3. The Significance of the Study

There is a dearth of data on the species composition of spontaneous vegetation growing on the mineral substrate of the hard coal heaps and the re-vegetation mechanisms of such sites. It is difficult to generalize about the processes mentioned above, which might be due to the enormous variety of non-analogous plant species composition in particular stages of spontaneous succession [10]. The post-hard coal mine heaps provide a number of heterogeneous microhabitats which are crucial for the coexistence of species with varied traits responding to the habitat’s requirements [17,44,79,80].

The obtained results supplement the knowledge about the relation between the vegetation species composition and the AMF parameters on the post-hard coal mine heaps. The importance of the knowledge is becoming more important, as the area transformed by various types of heaps, including those created by mining activities, covers almost 1% of the world’s land [80], and a wide range influences the environmental functioning [10,27,34,81,82,83].

Currently, the obligatory reclamation work often fails [37] or results in plant cover that can be sustained only at systematic interventions and high expense [84]. Moreover, the obligatory reclamation work frequently reduces the spontaneous biodiversity [80]. This kind of reclamation fails because it ignores the ecological principles, mainly reciprocal relationships between abiotic and biotic environments and the complex newly established relations between different groups of organisms [81,85,86,87,88]. In contrast, spontaneous succession is cheap, and spontaneously re-vegetated sites usually exhibit a higher natural value as a result of significant biodiversity [80,84,89,90].

Our study showed that all mycorrhizal parameters were lower for *Calamagrostis epigejos* compared to *Poa compressa* in all studied vegetation patches, representing the chronosequence of the novel ecosystem succession stages developing on mineral soilless substrate. Mycorrhizal status of *Poa compressa* and *Calamagrostis epigejos* differed depending on the dominant plant species in the sampled vegetation patch. Against our expectations, the heap’s age was not the factor driving the intensity of mycorrhiza parameters in the studied plant species. The vegetation type and the dominant plant species are the main factors determining the intensity of mycorrhiza colonization. The M% and A% parameters were the highest for *Calamagrostis epigejos* (not statistically significant) and *Poa compressa* (statistically significant) in vegetation patches in which *Daucus carota* was the dominant plant. Regardless of the harsh habitat conditions, the biotic influence of the co-occurring plant species influenced the AMF colonization.

## Figures and Tables

**Table 1:** The experiment design scheme.

Age of Heap	Dominant Plant Species		Tested Individuals	
			*P. compressa*	*C. epigejos*
Class I	*P. compressa*	four paches	40	
Class I	*P. compressa*	three paches		30
Class II	*C. epigejos*	five paches	50	
Class II	*D. carota*	four paches	40	
Class II	*C. epigejos*	four paches		40
Class II	*D. carota*	three paches		30
Class III	*B. pendula B*	two paches		20
Class III	*P.sylvestris B*	two paches		20
Class IV	*B. pendula A*	three paches	30	
Class IV	*B. pendula A*	three paches		30

Letter “*A”* after the species name indicates the tree form, letter “*B”* after species name indicates the shrub form, and species name without letter indicates the herb layer. Class I—up to 10 years old; Class II—10–30 years old; Class III—30–60 years old; Class IV—over 60 years old heap.

**Table 2:** Physico-chemical properties of the substrate from the rhizosphere zone of the target species growing in the studied vegetation patches on coal mine heaps.

Heap Age Class	Vegetation Type Sampled	The Studied Species Indivi-duals	pH (KCl)	P mg 100 g^−1^	K mg 100 g^−1^	Mg mg 100 g^−1^	orgC %	N-NH4 mg 100 g^−1^	Conductivity µS cm^−1^
Class I	*Poa compressa*	*C. epigejos*	6.47 ± 0.18	3.57 ± 0.77	13.97 ± 3.62	26.67 ± 3.06	10.00 ± 0.46	3.28 ± 2.70	175.00 ± 32.65
Class II	*Calamagrostis epigejos*	*C. epigejos*	5.51 ± 0.68	4.02 ± 0.22	15.63 ± 1.65	32.50 ± 3.73	10.10 ± 0.74	1.38 ± 0.20	136.00 ± 21.72
Class II	*Daucus carota*	*C. epigejos*	5.73 ± 0.49	6.60 ± 0.61	16.13 ± 5.48	16.27 ± 7.23	15.63 ± 6.82	3.10 ± 0.49	82.67 ± 5.36
Class III	*Betula pendula B*	*C. epigejos*	5.30 ± 0.89	6.20 ± 1.65	17.83 ± 0.62	22.27 ± 3.56	9.00 ± 1.72	2.50 ± 0.78	111.00 ± 21.38
Class IV	*Betula pendula A*	*C. epigejos*	4.60 ± 0.18	4.77 ± 1.33	9.87 ± 2.55	10.97 ± 5.40	7.57 ± 3.58	2.74 ± 1.35	74.33 ± 9.84
Class III	*Pinus sylvestris B*	*C. epigejos*	6.75 ± 0.07	4.70 ± 0.85	4.15 ± 0.50	15.10 ± 1.98	18.70 ± 0.57	5.92 ± 0.54	123.00 ± 18.38
Class I	*Poa compressa*	*P. compressa*	6.27 ± 0.17	4.08 ± 0.32	15.70 ± 0.34	32.00 ± 2.02	6.90 ± 0.44	1.86 ± 0.20	166.50 ± 23.09
Class II	*Calamagrostis epigejos*	*P. compressa*	4.59 ± 0.62	5.60 ± 0.95	16.05 ± 4.60	18.88 ± 4.25	13.22 ± 5.86	3.46 ± 0.97	242.30 ± 78.59
Class II	*Daucus carota*	*P. compressa*	6.23 ± 0.36	4.94 ± 0.72	19.50 ± 2.22	27.68 ± 2.72	7.38 ± 1.21	2.93 ± 1.08	198.00 ± 97.75
Class IV	*Betula pendula A*	*P. compressa*	6.55 ± 1.13	4.40 ± 1.76	13.47 ± 0.18	23.67 ± 4.76	10.00 ± 3.63	2.60 ± 0.74	73.00 ± 3.79

Results are means ± SE (*n* = 35); orgC—organic carbon. Letter *“A”* after the species name indicates the tree form, letter *“B”* after species name indicates the shrub form, species name without a capital letter indicates the herb layer.

**Table 3:** The inclusion of analyzed factors in the RDA model showing their impact on root colonization of studied plants.

Code	Description	Df	AIC	Pseudo-F	*p*-Value
Mg	Mg (mg/100 g)	1	240.56	4.3205	0.015
K	K_2_O (mg/100 g)	1	241.78	3.0345	0.020
Age	Mg (mg/100 g)	1	241.36	3.4667	0.025
DCA1	DCA axis 1	1	241.82	2.9982	0.035
CE	*Calamagrostis epigejos*	1	243.54	1.2636	NS
PC	*Poa compressa*	1	243.54	1.2636	NS
DCA2	DCA axis 2	1	243.62	1.1863	NS
Year	Year of study	1	244.14	0.6832	NS
P	P_2_O_5_ (mg/100 g)	1	244.36	0.4712	NS
pH	pH	1	244.69	0.1569	NS

**Table 4:** The summary of the main results of the study conducted on *Calamagrostis epigejos* and *Poa compressa* and AMF colonization.

Species	Soil	Spoil	Method	AMF	References
*Calamagrostis epigejos*	loess, perlite, Czech Republik	coal mine	Trouvelot	94%	[55]
	loess, clay, perlite, Czech Republik	coal mine	Trouvelot	80%	[55]
	clay, Czech Republik	contains fly ash from a power station burning brown coal	Giovanetti and Mosse	present, no data	[68]
	loess, Czech Republik	coal mine	Trouvelot	75–97%	[69]
	gravel, sand, loess,	fireloam strip mine	McGonigle et al.	present, no data	[66]
	caolinite-montmorilonite-illite clays	industral areas	Giovanetti and Mosse	present, no data	[52]
	clay, Czech Republik	coal mine	Giovanetti and Mosse	present	[50]
*Poa compressa*	calcareous slope with thin-layered rendzina soil	exposed to emissions of a nearby phosphate fertilizer factory	McGonigle et al.	present 12–42%	[58]
	peat-vermiculite	-	McGonigle et al.	present	[70]
	clay	-	-	40%	[71]
	mulched	coal mine	McGonigle et al.	5–95%	[72]
	clay, rocks	serpentine open-pit mine	McGonigle et al.	present	[73]

## Data Availability

No new data were created or analyzed in this study. Data sharing is not applicable to this article.
